# Smooth muscle cell recruitment to lymphatic vessels requires PDGFB and impacts vessel size but not identity

**DOI:** 10.1242/dev.147967

**Published:** 2017-10-01

**Authors:** Yixin Wang, Yi Jin, Maarja Andaloussi Mäe, Yang Zhang, Henrik Ortsäter, Christer Betsholtz, Taija Mäkinen, Lars Jakobsson

**Affiliations:** 1 Karolinska Institutet, Department of Medical Biochemistry and Biophysics, Division of Vascular Biology, Scheeles Väg 2, SE171 77 Stockholm, Sweden; 2 Uppsala University, Dept. Immunology, Genetics and Pathology, Rudbeck Laboratory, Dag Hammarskjölds väg 20, SE751 85 Uppsala, Sweden; 3 Integrated Cardio Metabolic Centre (ICMC), Karolinska Institutet, Novum, Blickagången 6, SE14157 Huddinge, Sweden

**Keywords:** Lymphatic vasculature, PDGFB, Contraction, Lymphedema, Morphogenesis, Smooth muscle cell

## Abstract

Tissue fluid drains through blind-ended lymphatic capillaries, via smooth muscle cell (SMC)-covered collecting vessels into venous circulation. Both defective SMC recruitment to collecting vessels and ectopic recruitment to lymphatic capillaries are thought to contribute to vessel failure, leading to lymphedema. However, mechanisms controlling lymphatic SMC recruitment and its role in vessel maturation are unknown. Here, we demonstrate that platelet-derived growth factor B (PDGFB) regulates lymphatic SMC recruitment in multiple vascular beds. PDGFB is selectively expressed by lymphatic endothelial cells (LECs) of collecting vessels. LEC-specific deletion of *Pdgfb* prevented SMC recruitment causing dilation and failure of pulsatile contraction of collecting vessels. However, vessel remodelling and identity were unaffected. Unexpectedly, *Pdgfb* overexpression in LECs did not induce SMC recruitment to capillaries. This was explained by the demonstrated requirement of PDGFB extracellular matrix (ECM) retention for lymphatic SMC recruitment, and the low presence of PDGFB-binding ECM components around lymphatic capillaries. These results demonstrate the requirement of LEC-autonomous PDGFB expression and retention for SMC recruitment to lymphatic vessels, and suggest an ECM-controlled checkpoint that prevents SMC investment of capillaries, which is a common feature in lymphedematous skin.

## Abstract (precis)

**Summary:** *Pdgfb* mutant mice provide insight into the recruitment and function of smooth muscle cells in the lymphatic vasculature, and shed new light on mechanisms of lymph vessel-associated diseases.

## INTRODUCTION

The lymphatic vasculature in mammals controls homeostasis by transporting liquid from tissue back to the blood circulation. It is also part of the immune system and filters the lymph through lymph nodes for immune surveillance. The lymphatic vasculature is organised in a hierarchy of capillaries, pre-collectors and collecting lymphatic vessels, each with specific functional and morphological features (Lutter et al., 2012; Ulvmar and Mäkinen, 2016). Lymphatic capillaries lack mural cell coverage and are blind-ended vessels with permeable button-like cell junctions, which allow fluid uptake and immune cell entry from the tissue (Baluk et al., 2007; Pflicke and Sixt, 2009). Downstream of the capillaries are the pre-collectors, which are sparsely covered by smooth muscle cells (SMCs) and drain into the collecting vessels with more extensive SMC coverage. Here, valves and continuous zipper-like endothelial cell junctions ensure an efficient unidirectional lymph transport (Alitalo, 2011; Baluk et al., 2007; Koltowska et al., 2013; Schulte-Merker et al., 2011; Ulvmar and Mäkinen, 2016).

Impaired lymphatic drainage causes accumulation of fluid in the tissue and leads to swelling, referred to as lymphedema. Long-term symptoms include discomfort, pain and an increased incidence of infections (Williams et al., 2005). Lymphedema can be caused by genetic mutations (primary), or trauma, infections, cancer surgery and/or irradiation (secondary); however, knowledge of the pathological mechanisms is still limited (Szuba and Rockson, 1998). Collecting lymphatic vessels and their valves are known to be crucial for proper lymph drainage but the role of SMCs in this process is only partly understood. For example, SMC contraction has been shown to promote lymph drainage in the larger collecting vessels, such as the popliteal vessels (Kunert et al., 2015; Liao et al., 2011), but its importance for the function of smaller collecting vessels like those present in the skin, a tissue commonly involved in lymphedema, is not known. Nevertheless, deficient SMC function has been suggested to be an integral part of chronic lymphedema in humans (Ogata et al., 2015). In individuals suffering from lymphedema distichiasis (LD), which is caused by mutations in the forkhead transcription factor *FOXC2*, lymphedema is accompanied by valve agenesis but also by aberrant recruitment of SMCs to lymphatic capillaries (Petrova et al., 2004). Genetic deletion of *Foxc2* as well as of *Ang2* or *Efnb2* in mice recapitulates this disease phenotype and leads to profound lymphatic remodelling defects that are characterised by defective valve formation, SMC recruitment and establishment of collecting vessel and capillary lymphatic endothelial cell (LEC) identities (Gale et al., 2002; Makinen et al., 2005; Norrmén et al., 2009; Petrova et al., 2004; Sabine et al., 2015). Altogether, these studies suggest an important role for SMC and LEC interplay in lymphatic morphogenesis and function, but precisely how SMCs regulate these processes is not known.

The successive steps of SMC recruitment to the lymphatic vasculature during development have been characterized in both dermal and mesenteric lymphatic vessels (Lutter et al., 2012; Norrmén et al., 2009). In the mouse ear, SMCs start to colonize the collecting vessels at postnatal day (P) 14, which coincides with downregulation of lymphatic vessel hyaluronan receptor 1 (LYVE-1). From P16, only lymphatic capillaries maintain *Lyve1* expression and also remain devoid of SMCs throughout life. The reciprocal cellular interaction between SMCs and LECs has thus been suggested to regulate the establishment of capillary versus collecting vessel identity. Supporting this notion, SMC-LEC contact induces secretion and activation of the extracellular matrix glycoprotein reelin (Reln) specifically in LECs of collecting vessels, which in turn promotes further SMC recruitment (Lutter et al., 2012). In *Reln*^−/−^ mice, reduced SMC coverage is accompanied by sustained LYVE-1 expression in collecting vessels. Platelet-derived growth factor B (PDGFB), which is central for mural cell (SMCs and pericytes) recruitment to the blood vasculature (Hellstrom et al., 1999), is also expressed by LECs *in vivo* and may be similarly involved in recruitment of SMCs to lymphatic vessels (Tammela et al., 2007). For example, mesenchymal overexpression of PDGFB was shown to lead to SMC dissociation both from lymphatic vessels and veins (Tammela et al., 2007). In addition, upregulation of PDGFB in lymphatic capillaries of individuals with LD, which is proposed to be a consequence of loss of a direct FOXC2 suppressive effect, was suggested to underlie their ectopic SMC coverage (Meinecke et al., 2012; Petrova et al., 2004; Tammela et al., 2007). However, FOXC2 is highly expressed in developing collecting vessels to which SMCs are recruited. Hence FOXC2-mediated inhibition of PDGFB cannot explain the differential SMC recruitment to collecting vessels and capillaries, at least not during development. Altogether, these data suggest that SMC recruitment is important for the maturation of vessels into functional collecting vessels, nevertheless the direct role of SMCs in this process is not known.

Herein, we reveal that expression of *Pdgfb* within the developing dermal lymphatic vasculature is restricted to LECs of collecting vessels but absent from capillaries. Using conditional loss- and gain-of-function models, we show that LEC expression of PDGFB is required for SMC recruitment to collecting vessels, but that overexpression is insufficient to mediate recruitment to lymphatic capillaries. We find that in addition to PDGFB expression the ligand relies on its binding and retention to the local extracellular matrix to promote SMC recruitment. We also show that although SMCs are crucial for pulsatile contraction of collecting vessels, they are not required for dermal lymphatic vessel remodelling, valve morphogenesis and establishment of capillary versus collecting vessel identity.

## RESULTS

### PDGFB is selectively expressed by LECs of SMC covered collecting vessels but not by capillary LECs

**Fig. 1.**
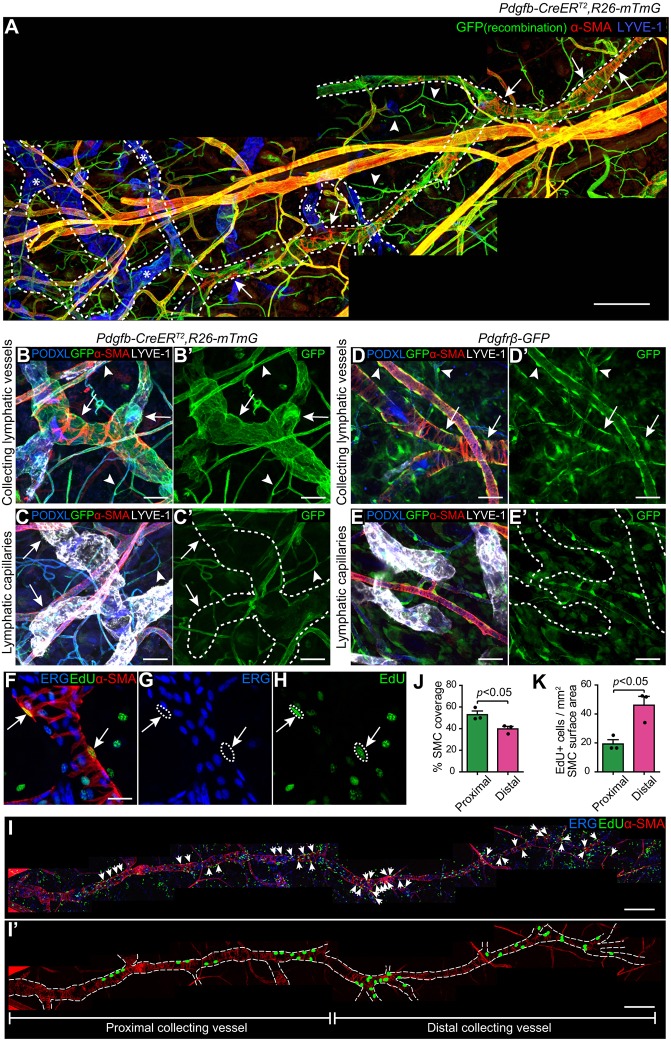
***Pdgfb* is expressed by collecting but not capillary LECs, and collecting vessel SMCs express PDGFRβ.** (A-C′) Immunostained dorsal ear skin of *Pdgfb-CreER^T2^,R26-mTmG* mice induced by 4-OHT at P14 and analysed at P21. (A) Overview of the lymphatic ear vasculature, outlined by dashed lines, including the SMC-covered (α-SMA+, red) collecting vessels (green, arrows) and the non-covered lymphatic capillaries (blue, asterisks). GFP indicates Cre-mediated recombination, as a consequence of an active *Pdgfb* promoter, observed in collecting vessels (arrows) and blood vessels (capillaries, arrowheads). (B,B′) Collecting vessels (podocalyxin^+^, LYVE-1^−^) are GFP positive (green, arrows) and are covered by SMCs (red). (C,C′) Lymphatic capillaries (LYVE-1^+^, white) are GFP negative (arrows, no expression of *Pdgfb*) and lack SMC coverage. (D-E′) Dorsal ear skin of *Pdgfrβ-GFP* mice at P21 stained for podocalyxin (blue), α-SMA (red) and LYVE-1 (white). GFP (green) positivity reveals the presence of *Pdgfrb-*expressing SMCs around collecting vessels (D,D′, arrows) and blood vessels (D,D′, arrowheads) but not in lymphatic capillaries (E,E′, dashed lines). (F-I′) Whole-mount staining of P21 mouse ear after EdU injection at P14, P16 and P18 indicates proliferating SMCs (arrows) on the lymphatic vessels with antibodies against ERG 1 (blue), EdU (green) and α-SMA (red). (I,I′) Distribution of EdU^+^ SMCs (I, arrows in upper panel; I′, schematic green ellipse in lower panel) in a complete collecting vessel. (J,K) Quantification of EdU^+^ and α-SMA^+^ cells (K) relating to SMC coverage (J) in the proximal and distal part of the entire collecting vessel (*n*=3). Data are mean±s.e.m. *P* value is calculated using Student's *t*-test. Scale bars: A, 200 µm; B-E′, 50 µm; F-H, 20 µm; I,I′, 200 µm.

SMCs cover arteries and veins of the blood vasculature, as well as the collecting lymphatic vessels (Fig. 1A). PDGFB is known to regulate mural cell recruitment to blood vessels but its potential involvement in the analogous process in the lymphatic vasculature has not been demonstrated. To characterize the precise cellular source of PDGFB within the developing vasculature, we used a double transgenic mouse (*Pdgfb-CreER^T2^-IRES-egfp;R26-mTmG*) carrying a *Pdgfb*-promoter-driven tamoxifen-inducible Cre-recombinase, a *Pdgfb*-driven GFP, as well as a conditional allele (loxp flanked stop) for inducible expression of a membrane-bound GFP functioning as a lineage tracer (Claxton et al., 2008; Muzumdar et al., 2007). Tamoxifen administration at P14 and analysis by immunostaining for GFP at P21 allowed for identification of cells expressing *Pdgfb* at either time point, as well as the progeny of PDGFB^+^ cells in P14 ears. In the mouse ear skin, GFP immunoreactivity revealed that *Pdgfb* was expressed throughout the blood vasculature, whereas in the lymphatics it was restricted to LECs of collecting vessels and absent in capillaries (Fig. 1A-C′). The spatial distribution of SMCs within the lymphatics inversely correlated with the expression of the lymphatic capillary marker LYVE-1 (Fig. 1A-C′). To relate the expression of the PDGFB ligand to the localisation of cells expressing its main receptor (PDGFRβ), we used a transgenic reporter mouse line with *Pdgfrb* promoter-driven expression of GFP (here denoted *Pdgfrβ-GFP*). Staining of the ear skin of P21 mice revealed colocalisation of GFP and α-smooth muscle actin (α*-*SMA), indicative of *Pdgfrb*-expressing SMCs in collecting vessels (Fig. 1D,D′). No GFP signal was observed in LYVE-1^+^ lymphatic capillaries, indicating the absence of other potential α*-*SMA^−^*;Pdgfrβ-GFP^+^* mural cells, such as pericytes (Fig. 1E,E′). These results demonstrate that, unlike the ubiquitous expression of *Pdgfb* in the blood vasculature, *Pdgfb* is restricted to collecting vessels within the lymphatic vasculature. The spatial correlation between LECs producing PDGFB and mural cells expressing PDGFRβ suggests that this ligand-receptor pair is involved in lymphatic SMC recruitment.

In addition to recruitment, SMC *in situ* proliferation may contribute to the final coverage of developing collecting lymphatic vessels. To investigate this, we administered EdU to wild-type mice, starting at the time of initial SMC recruitment (P14), and continued at P16 and P18 to cover the time period of collecting vessel maturation. Analyses of SMC coverage and proliferation at P21 (Fig. 1F-K) showed EdU^+^ SMCs along the entire length of collecting vessels. Although SMC coverage was higher in the proximal than in the distal ends of vessels, the proximal regions showed a relatively lower degree of SMC proliferation (Fig. 1I-K), suggesting that proliferation ceases as vessels mature. Interestingly, within the distal parts of the vessels EdU^+^ SMCs were confined to regions near vessel branches (Fig. 1I,I′). Together, these data suggest that SMCs not only populate the lymphatics by initial recruitment but also by subsequent proliferation.

### Postnatal LEC-specific deletion of *Pdgfb* prevents SMC recruitment to dermal collecting lymphatics, causing vessel dilation without affecting vessel hierarchy

**Fig. 2.**
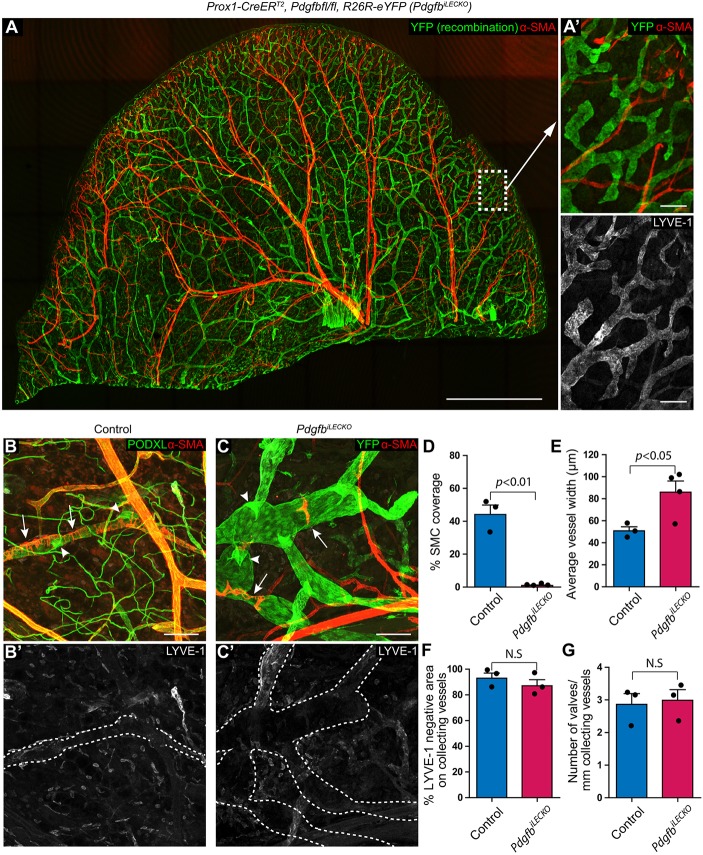
**Postnatal LEC-specific deletion of Pdgfb causes severe collecting vessel SMC deficiency without major effects on morphogenesis.** (A-C′) Whole-mount immunofluorescence of dorsal mouse ear skin of *Pdgfb^iLECKO^* mice (A,C,C′) and control mice (B,B′) at P21 stained for YFP (A,C, green), α-SMA (red), podocalyxin (B, green) and LYVE-1 (B′,C′, grey). Tiled confocal images (A) indicate successful recombination of the dermal lymphatic vasculature (YFP, green), including capillaries (A′, lower panel indicates LYVE-1 positivity) and collecting vessels (C). In *Pdgfb^iLECKO^* mice, only sporadic SMCs locate to collecting vessels (C, arrows) and the vessels are dilated compared with controls (B, arrows). Lymphatic valves appeared normal in the mutants (C, arrowheads) compared with the controls (B, arrowheads). Collecting vessel LYVE-1 downregulation and valve morphology are unchanged in the *Pdgfb^iLECKO^* mice (C′) compared with the controls (B′). (D,E) Quantification of collecting vessel SMC coverage (D) and vessel width (E), comparing the control mice (*n*=3) and the *Pdgfb^iLECKO^* mice (*n*=4). (F,G) Quantification of LYVE-1 negative area (F) and valve density (G) within collecting vessels of the *Pdgfb^iLECKO^* mice (*n*=3) compared with control mice (*n*=3). Scale bars: A, 2 mm; A′, 100 µm; B,C, 100 µm.

To investigate the role of LEC-autonomous PDGFB in the recruitment of SMCs, we generated *Prox1-CreER^T2^*; *Pdgfb^flox/flox^*; *R26R-eYFP* mice (henceforth denoted *Pdgfb^iLECKO^*). These mice allowed for tamoxifen-induced LEC-specific deletion of *Pdgfb*, as well as the identification of recombination by YFP expression. Daily tamoxifen administration from P4 to P7 and staining of the ear skin at P21 indicated specific recombination in the lymphatic vasculature, in accordance with previous data (Fig. 2A) (Bazigou et al., 2011). In addition, collecting lymphatic vessels of the *Pdgfb^iLECKO^* mice displayed a near-complete SMC deficiency (1.0% SMC coverage in *Pdgfb^iLECKO^* versus 44.2% in control) as revealed by image analysis (Fig. 2A,B,C,D; Fig. S1A,B). Immunolabelling for PDGFRβ illustrated the absence of other potential PDGFRβ^+^;α-SMA^−^ mural cell populations (Fig. S1E-F′). No apparent change in blood vascular morphogenesis or mural cell coverage was recorded (Fig. S1C,D). Furthermore the SMC-devoid lymphatic collecting vessels of the *Pdgfb^iLECKO^* mice displayed increased diameter (Fig. 2E), but showed no alteration in lymphatic capillary morphology. In the absence of SMCs in the *Pdgfb* mutant, LYVE-1 was downregulated in the collecting vessels to a similar degree to controls (Fig. 2B′,C′,F). In addition, the number and architecture of lymphatic valves were unchanged in the *Pdgfb^iLECKO^* mice compared with controls (Fig. 2G). We further assessed mural cell coverage of collecting vessels following induced deletion of *Pdgfb* using the *Cdh5(PAC)-CreER^T2^* mouse that targets both blood and lymphatic vessels (Wang et al., 2010). Tamoxifen induction and analysis of *Cdh5(PAC)-CreER^T2^*; *Pdgfb^flox/flox^*; *R26R-eYFP* (*Pdgfb^iECKO^*) mice revealed recombination (YFP^+^) in both blood and lymphatic endothelial cells (Fig. S2D). Mural cell coverage of blood vessels was not drastically affected; however, SMC coverage of lymphatic collecting vessels was reduced (Fig. S2A,B,C), thereby recapitulating the observations from the *Pdgfb^iLECKO^* mice. The lack of an obvious effect on blood vessel SMC coverage may reflect a higher degree of maturation of the blood vasculature compared with the lymphatic vessels at the time of induction. Together, these data provide direct genetic evidence for the requirement of LEC-derived *Pdgfb* in the recruitment of mural cells to the lymphatic collecting vessels and indicate that SMCs are not required for lymphatic vessel remodelling into a hierarchy of collecting vessels and capillaries.

### Establishment of collecting versus capillary LEC identities does not require SMC interaction

**Fig. 3.**
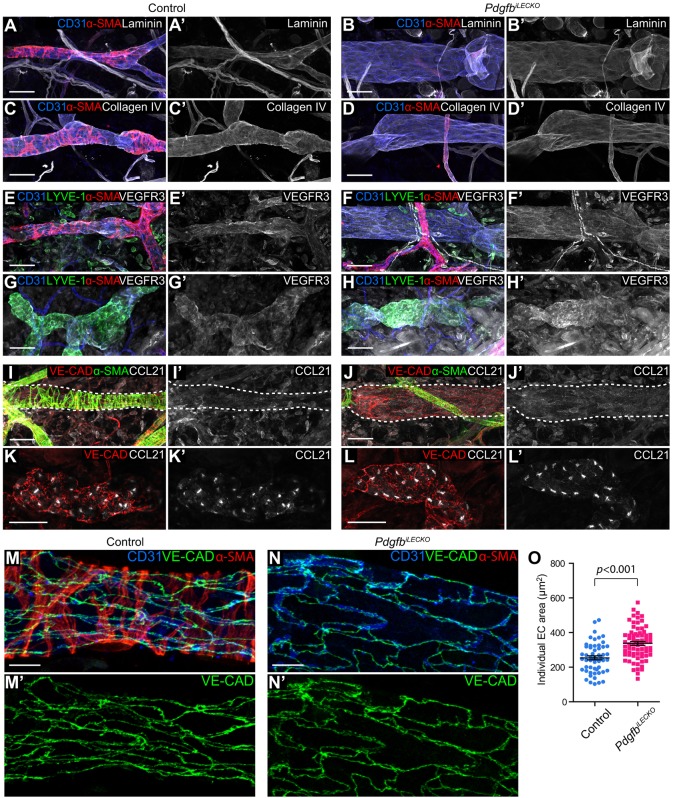
**Absence of perivascular SMCs does not affect the establishment of LEC identities.** Whole-mount immunofluorescence of dorsal mouse ear skin of control (left) or *Pdgfb^iLECKO^* (right) mice at P21. (A-B′) Antibodies against CD31 (blue), α-SMA (red) and laminin (grey). (C-D′) Antibodies against CD31 (blue), α-SMA (red) and collagen IV (grey). (E-H′) Antibodies against CD31 (blue), LYVE-1 (green), α-SMA (red) and VEGFR3 (grey). (I-L′) Antibodies against VE-CAD (red), α-SMA (green) and CCL21 (grey). Collecting vessels are indicated by dashed lines (I-J′). None of the above proteins were altered following *Pdgfb* deletion. (M-N′) Immunostaining of mouse ear skin with antibodies against CD31 (blue), VE-CAD (green) and α-SMA (red). Individual LEC size is enlarged on the collecting vessel of *Pdgfb^iLECKO^* mice compared with the control mice. (O) Quantification of individual cell area of LECs (*n*=72) from two *Pdgfb^iLECKO^* mice and LECs (*n*=55) from two control mice. Data are mean±s.e.m. and *P* value is calculated using Student's *t*-test. Scale bars: A-L′, 50 µm; M-N′, 10 µm.

The basement membrane (BM) of collecting vessels is continuous, whereas that of capillaries is described as discontinuous (Pflicke and Sixt, 2009). To further investigate the potential involvement of SMCs in the maturation of collecting vessels, we studied the abundance of BM proteins in lymphatic vessels of the *Pdgfb^iECKO^* mice. Immunostaining for laminin (Fig. 3A-B′) and collagen IV (Fig. 3C-D′) revealed no differences between dermal lymphatic vessels in the *Pdgfb^iECKO^* and control ears in either intensity or continuity. In addition, VEGFR3, a ubiquitous LEC marker, was unchanged in both collecting vessels (Fig. 3E-F′) and capillaries (Fig. 3G-H′). The expression pattern of the capillary-restricted CCL21 as well as the junction protein vascular endothelial cadherin (VE-CAD) was unaffected in the *Pdgfb^iLECKO^* mice (Fig. 3J,J′,L,L′) compared with control mice (Fig. 3I,I′,K,K′). However, despite normal levels and junctional localisation of VE-CAD in LECs of collecting vessels of *Pdgfb^iLECKO^* mice, individual cells were enlarged compared with LECs from control mice (Fig. 3M-O). Together, these data demonstrate that the establishment of lymphatic vessel identity and vascular hierarchy is independent of SMC interaction but that such interaction affects cellular size. They furthermore indicate that the contribution of SMCs to the major constituents of the BM of collecting vessels is minor.

### PDGFB is required for SMC recruitment to the large-diameter collecting vessels of the hind limb and mesentery

**Fig. 4.**
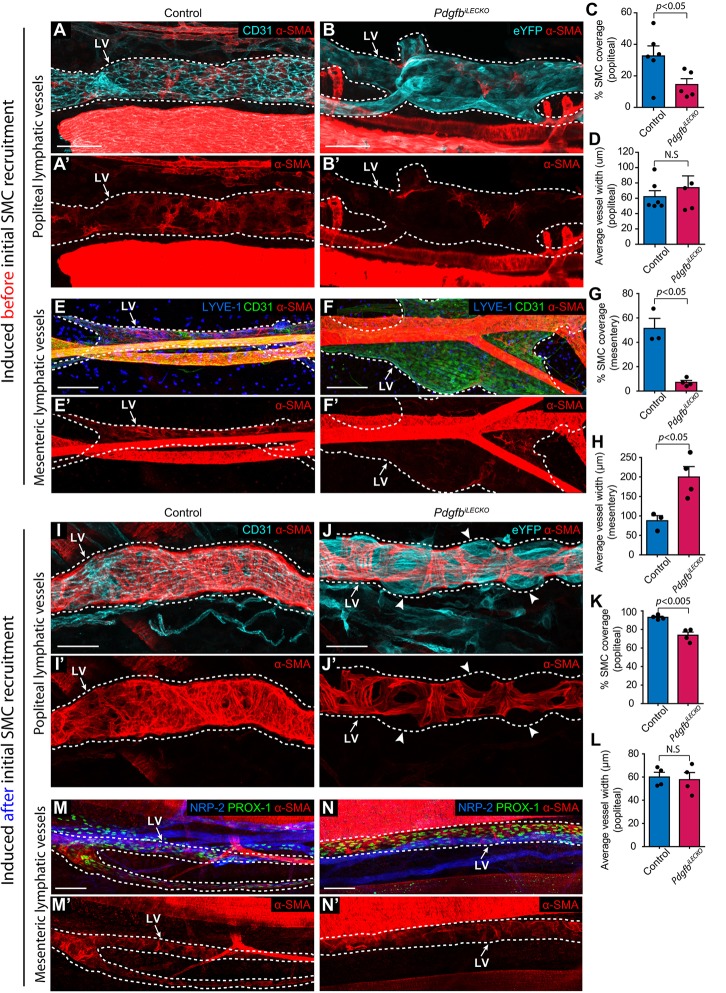
**SMC recruitment to large diameter vessels requires PDGFB.** Whole-mount immunofluorescence of popliteal and mesenteric collecting vessels from control mice and *Pdgfb^iLECKO^* mice with PDGFB deletion induced either before (induced at E15, E16 and E17) or after (induced at P1 and P2) initial SMC recruitment. (A-B′) Popliteal vessels of E18.5 embryos following induction at E15, E16 and E17. Immunostaining of lymphatic vessels (LV, dashed line) with antibodies against CD31 (A, cyan), YFP (B, cyan) and α-SMA (red). (C,D) Quantification of SMC coverage (C) and average vessel width (D) comparing control embryos (*n*=6) and *Pdgfb^iLECKO^* embryos (*n*=5). (E-F′) Mesenteric lymphatic vessels of E18.5 embryos following induction at E15, E16 and E17. Immunostaining of lymphatic vessels (LV, dashed line) with antibodies against LYVE-1 (blue), CD31 (E, green), YFP (F, green) and α-SMA (red). (G,H) Quantification of SMC coverage (G) and average vessel width (H) comparing control embryos (*n*=3) and *Pdgfb^iLECKO^* embryos (*n*=4). (I-J′) Popliteal vessels of P12 mice, induced by topical tamoxifen treatment at P1 and P2. Immunostaining of lymphatic vessels (LV, dashed line) with antibodies against CD31 (I, cyan), YFP (J, cyan) and α-SMA (red). Vessel segments lacking SMC coverage in *Pdgfb^iLECKO^* mice were enlarged (J,J′, arrowheads). (K,L) Quantification of SMC coverage (K) and average vessel width (L) comparing control mice (*n*=4) and *Pdgfb^iLECKO^* mice (*n*=4). (M-N′) Mesenteric lymphatic vessels of P12 mice treated with tamoxifen at P1 and P2. Immunostaining of lymphatic vessels (LV, dashed line) with antibodies against NRP-2 (blue), PROX-1 (green) and α-SMA (red). Data are mean±s.e.m. *P* value is calculated using Student's *t*-test. Scale bars: A-B′, 100 µm; E-F′, 200 µm; I-J′, 50 µm; M-N′, 100 µm.

Recruitment of mural cells to larger diameter collecting vessels, such as the popliteal and mesenteric collecting vessels, occurs during embryogenesis. To study the involvement of PDGFB in these tissues, we deleted PDGFB in *Pdgfb^iLECKO^* embryos by administration of 4-OHT to pregnant females at embryonic day (E)15, E16 and E17, and analysed the embryos at E18.5. SMC coverage in the *Pdgfb^iLECKO^* mice was reduced in both popliteal (Fig. 4A-C) and mesenteric lymphatic vessels (Fig. 4E-G) compared with controls. Although the diameter of popliteal lymphatic vessels was unaltered (Fig. 4D), mesenteric vessels were enlarged (Fig. 4H), suggesting tissue-specific consequences of SMC reduction. To investigate whether PDGFB is also required for expansion and maintenance of SMCs after their initial recruitment, PDGFB deletion was induced at P1 and P2 and lymphatic vessels were compared between *Pdgfb^iLECKO^* and control mice at P12. SMC coverage of the popliteal vessels was reduced to 73.8% compared with 93.1% in the control (Fig. 4I-K), while it remained unchanged in the mesenteric vessels (Fig. 4M-N′). Interestingly, local regions lacking SMC coverage in the popliteal vessels of *Pdgfb^iLECKO^* were bulging, demonstrating local effects of SMCs in constriction of the vessel (Fig. 4J,J′, arrowheads). Nevertheless the average vessel diameter remained unchanged (Fig. 4L). Together, these data reveal the strict requirement of LEC-derived PDGFB in SMC recruitment to large diameter collecting vessels of the hind limb and mesentery.

### Loss of SMCs causes impaired contraction of dermal collecting vessels

**Fig. 5.**
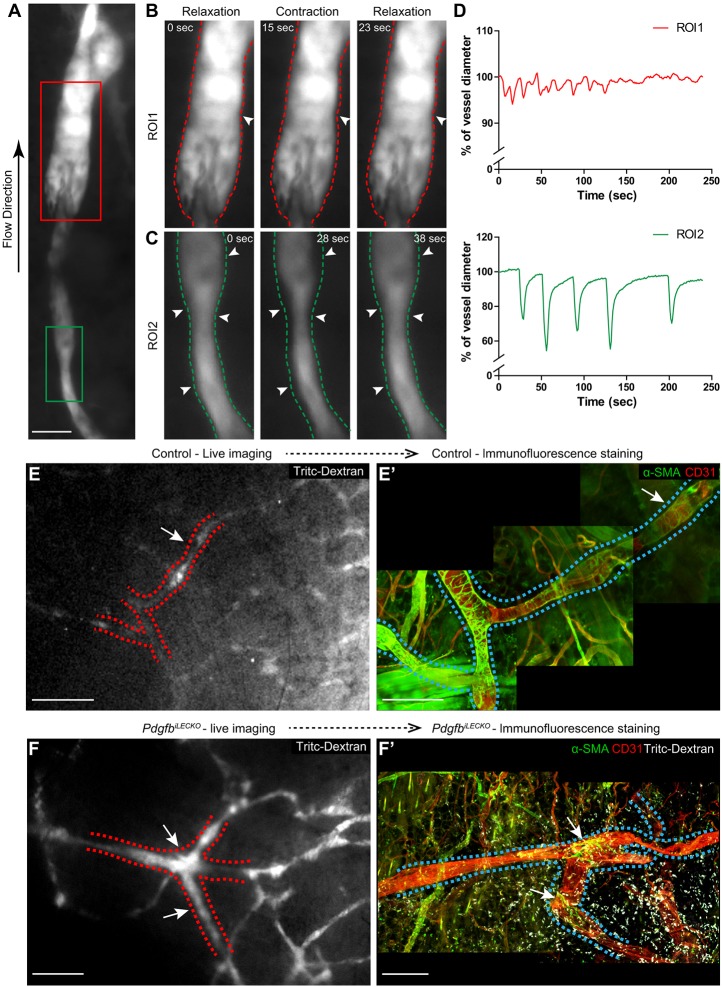
**Pulsatile contraction of dermal lymphatic collecting vessels requires SMC coverage.** (A-C) Lymphatic collecting vessels of ears of living mice, highlighted by subcutaneous injections with Tritc-dextran (grey). Snap shots from live-imaging of lymphatic collecting vessels analysed as two regions of interest (A, red box and green box). Snap shots of vessels contracting (arrowheads) and relaxing in the respective region of interests (ROIs) (ROI1, red box, magnified in B; ROI2, green box, magnified in C). (D) Relative change in vessel diameter over time for ROI1 (B) and ROI2 (C) reflects regional contraction patterns. (E,F) Snap shots from movies of contracting vessels in ears of control (E) or *Pdgfb^iLECKO^* (F) mice. (E',F') Whole-mount immunofluorescent staining of the live-imaged vessels in E and F. Areas of contraction (dashed line) were covered by SMCs in control mice (arrows, green in E′; intensity adjustments were applied specifically to the vessel area in order to enhance the visibility of perivascular SMCs) as well as in the only two areas of the *Pdgfb^iLECKO^* mouse (arrows, green in F′). Scale bars: A, 20 µm; E,F, 500 µm; E′,F′, 200 µm.

Despite the near-complete absence of dermal lymphatic SMC coverage in the *Pdgfb^iLECKO^* mice, no obvious lymphedema could be observed. Although contraction of large diameter vessels, such as popliteal and mesenteric lymphatic vessels, has been previously documented, dermal vessel contraction remains poorly studied and has not been correlated with SMC function or presence (Liao et al., 2011; Sabine et al., 2015). To further assess potential effects of mural cell loss on lymphatic properties, we studied lymphatic vessel contraction and drainage following tracer injection, using non-invasive *in vivo* live imaging of the ear skin. Using high-frequency non-invasive imaging, pulsatile vessel contraction was recorded (Movie 1). Dermal collecting vessels displayed a wide range of contraction frequency and amplitude (Fig. 5A-D). Notably, only a subpopulation of collecting dermal lymphatic vessels was seen to contract under the experimental conditions applied. To investigate the dependence and role of SMCs on this contractile behaviour, *Pdgfb^iLECKO^* mice (*n*=6) and control mice (*n*=10) at an age of 1 month were imaged and compared. The number of contraction sites per ear was significantly lower in *Pdgfb^iLECKO^* mice compared with control mice (*P*<0.05, Mann–Whitney Test; Movies 2 and 3). Out of 10 control mice analysed, 32 contraction sites were observed in seven mice and the contraction sites were associated with SMC coverage, indicated by immunofluorescence staining of the same ear after live imaging (Fig. 5E,E′; Movie 4). However, only one out of six *Pdgfb^iLECKO^* mice displayed collecting vessel contraction, restricted to two sites. Importantly, immunofluorescent staining of this ear revealed local sparse SMC coverage that precisely matched the sites observed to contract during live imaging (Fig. 5F,F′; Movie 5). These observations demonstrate that SMCs are strictly required for pulsatile lymphatic vessel contraction. To test the functionality of the non-contracting and widened lymphatic vasculature, a fluorescent tracer was subcutaneously injected into the ear of *Pdgfb^iLECKO^* and control mice. However, live imaging did not reveal any difference in the clearance of the injected tracer from tissue over time (Fig. S3).

### Postnatal LEC-specific overexpression of PDGFB does not induce ectopic SMC recruitment to lymphatic capillaries

**Fig. 6.**
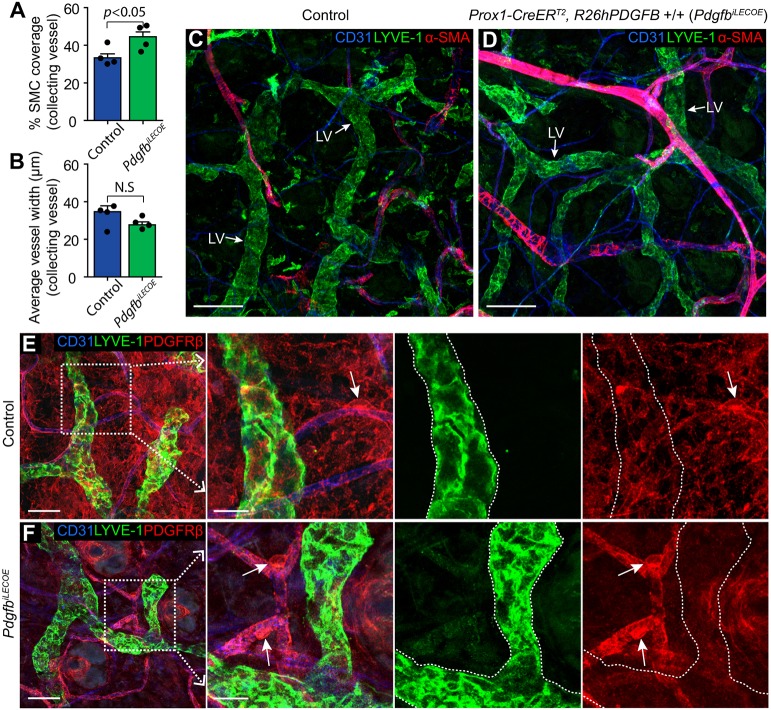
**Overexpression of PDGFB throughout the lymphatic vasculature does not cause aberrant SMC recruitment to capillaries.** (A,B) Quantification of SMC coverage (A) and average vessel width (B) of collecting vessels from control mice (*n*=5) and *Pdgfb^iLECOE^* mice (*n*=3) based on analyses of whole-mount immunofluorescent labelling of dorsal ear skin (images refer to Fig. S4B,C). Data are mean±s.e.m. *P* value is calculated using Student's *t*-test. (C,D) Whole-mount immunofluorescence staining of lymphatic vessels (LV, arrow) in the dorsal ear skin of control mice (C) and *Pdgfb^iLECOE^* mice (D) at 1 month of age with antibodies against CD31 (blue), LYVE-1 (green) and α-SMA (red). (E,F) Whole-mount immunofluorescence staining of lymphatic vessels (LV, arrow) in the dorsal ear skin of control mice (E) and *Pdgfb^iLECOE^* mice (F) at the age of 1 month, with antibodies against CD31 (blue), LYVE-1 (green) and PDGFRβ (red). Mural cells expressing PDGFRβ are found on blood vessels (arrows) but not on lymphatic capillaries (dashed line), despite PDGFB overexpression in *Pdgfb^iLECOE^* mice. Scale bars: C,D, 100 µm; E, 50 µm; E (high magnification), 20 µm; F, 50 µm; F (high magnification), 20 µm.

To investigate whether aberrant PDGFB expression within lymphatic capillaries is sufficient to induce SMC recruitment, we crossed *Prox1-CreER^T2^* mice with *R26*h*PDGFB*^+/+^ mice (Armulik et al., 2010), allowing for inducible LEC-specific overexpression of human PDGFB. Endothelial cell-specific expression of the *R26*h*PDGFB*^+/+^ allele has been demonstrated to rescue embryonic lethality and mural cell recruitment of *Pdgfb*^−/−^ mice (Armulik et al., 2010). *Prox1-CreER^T2^*; *R26*h*PDGFB*^+/+^ mice (*Pdgfb^iLECOE^*) and controls received three doses of tamoxifen between P2 and P8, followed by analysis of the dermal ear lymphatic vasculature at 4 weeks of age. *Prox1-CreER^T2^*-mediated recombination driving expression of the human PDGFB was observed throughout the lymphatic vasculature, as indicated by visualisation of the conditional reporter (data not shown; see Fig. 2A). Ectopic expression in lymphatic capillaries was confirmed by detection of human *PDGFB* transcripts in FACS-sorted LYVE-1+ LECs from dermal ear skin of *Pdgfb^iLECOE^* mice (Fig. S4A). SMC coverage of the collecting vessels of *Pdgfb^iLECOE^* mice was modestly increased compared with littermate controls (Fig. 6A) (44.5% versus 33.4%, *P*<0.05) illustrating functionality of induced overexpression, as well as a PDGFB dose effect on SMC recruitment. Collecting vessels in *Pdgfb^iLECOE^* mice further showed a trend towards reduced average diameter (Fig. 6B). However, despite aberrant PDGFB expression in capillary LECs, no α-SMA^+^ or PDGFRβ^+^ cells were recruited to lymphatic capillaries (Fig. 6C-F). These data demonstrate that, although PDGFB is essential for SMC recruitment to the lymphatic vasculature, LEC expression is not sufficient to mediate recruitment of SMCs to lymphatic capillaries.

### Deficient interaction between PDGFB and extracellular matrix causes defective SMC recruitment to dermal collecting lymphatic vessels

**Fig. 7.**
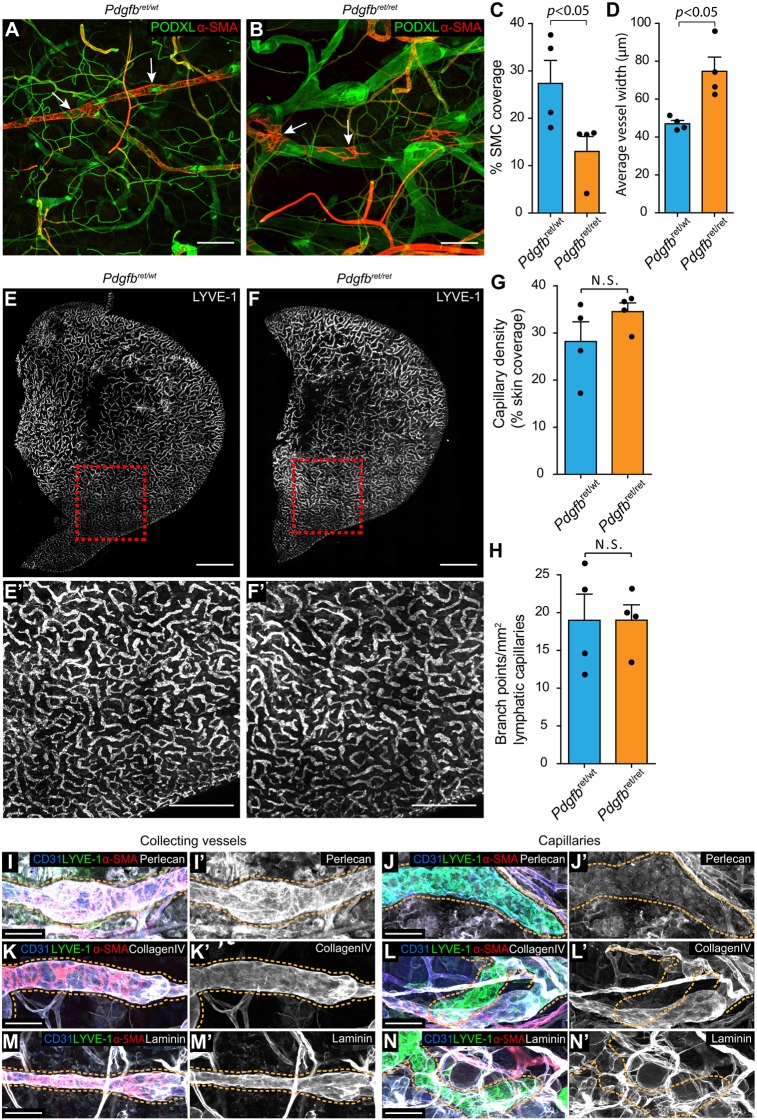
**Impaired PDGFB retention in the extracellular matrix causes defective SMC recruitment to collecting vessels without affecting lymphatic capillaries.** (A,B) Dorsal ear skin of mice at 10 weeks of age stained for podocalyxin (green) and α-SMA (red). SMCs form concentric rings in collecting vessels in *Pdgfb^ret/wt^* mice (A, arrows) but are only sparsely covering collecting vessel in *Pdgfb^ret/ret^* mice (B, arrows). (C,D) Quantification of SMC coverage (C) and average vessel width (D). (E-F′) Whole-mount immunofluorescence staining of capillaries of *Pdgfb^ret/wt^* mice (E; E′ at high magnification) and *Pdgfb^ret/ret^* mice (F; F′ at high magnification) with antibody against LYVE-1. (G,H) Quantification of lymphatic capillary density (G) and branch points (H) comparing *Pdgfb^ret/wt^* mice (*n*=4) and *Pdgfb^ret/ret^* mice (*n*=4). Data are mean±s.e.m. *P* value is calculated using Student's *t*-test. (I-N′) Dorsal ear skin of the control mouse at 1 month of age with antibodies against CD31 (blue), LYVE-1 (green), α-SMA (red), perlecan (I-J′, grey), collagen IV (K-L′, grey) and laminin (M-N′, grey) comparing the collecting vessels (I,I′,K,K′,M,M′, dashed line) and the capillaries (J,J′,L,L′,N,N′, dashed line). Scale bars: A,B, 200 µm; E,F, 2 mm; E′,F′, 1 mm; I-N′, 50 µm.

Mural cell recruitment to the blood vasculature relies not only on endothelial cell production of PDGFB but also on its binding to the extracellular matrix. To investigate whether such interaction is important also for the lymphatic vasculature, we studied mice with a genetically modified PDGFB that lack its heparan sulphate-binding domain (*Pdgfb*^ret/ret^) (Lindblom et al., 2003). Here, heterozygote *Pdgfb*^ret/wt^ served as controls as they showed no alterations of collecting vessels (Fig. 7A). Similar to the situation in *Pdgfb^iLECKO^* mice, *Pdgfb*^ret/ret^ mice displayed reduced SMC coverage and dilation of dermal ear lymphatic collecting vessels (Fig. 7A-D), without affecting density or number of branch points of the lymphatic capillaries (Fig. 7E-H). These results show that interaction between extracellular matrix and PDGFB is required for correct recruitment of SMCs to the lymphatic collecting vessels of the skin. Differential composition of the collecting versus capillary BMs, with respect to PDGFB-binding molecules, may thus contribute to the inability of ectopically expressed PDGFB to induce mural cell recruitment to the lymphatic capillaries. To investigate this, we assessed lymphatic vascular expression and deposition of heparan sulphate proteoglycan perlecan, as well as collagen IV, which are known to bind PDGFB either via the heparan sulphate chains or directly via the core protein (Gohring et al., 1998). Immunostaining for perlecan, collagen IV and laminin revealed high levels of both proteins in dermal collecting vessels (Fig. 7I,I′,K,K′,M,M′) but strikingly lower levels in capillaries (Fig. 7J,J′,L,L′,N,N′). These data suggest that, in the absence of PDGFB-binding BM proteins, PDGFB may not be sufficiently retained in close proximity to the LECs of capillaries to allow for recruitment of mural cells, even following ectopic PDGFB expression.

## DISCUSSION

Here, we generated novel mice allowing for inducible LEC-specific deletion of PDGFB. PDGFB deletion rendered the dermal lymphatic vasculature of the ear completely devoid of SMCs and led to reduced SMC coverage of mesenteric and popliteal vessels, in turn revealing a strict requirement for LEC-derived PDGFB in SMC recruitment to collecting vessels. Using this model, we provide the first description of the direct role of SMCs in lymph vessel morphogenesis and function, independent of other factors. The data indicate that several key aspects of lymphatic vascular development do not rely on SMCs, in contrast to previous suggestions.

SMC recruitment to the collecting vessels has been shown to coincide with vessel remodelling and maturation. Several studies have, in addition, revealed abnormal SMC coverage on lymphatic vessels in disease as well as in genetic mouse models. Some of these investigations have inferred an inverse correlation between SMC coverage and LYVE-1 expression (Dellinger et al., 2008; Lutter et al., 2012; Meinecke et al., 2012; Petrova et al., 2004; Yu et al., 2016). In support of the role of SMCs in promoting *Lyve1* downregulation, Tammela et al. showed that mesenchymal overexpression of PDGFB in the mouse ear skin resulted in displacement of collecting vessel SMCs, accompanied by increased expression of LYVE-1 (Tammela et al., 2007). In addition, reduced SMC coverage of *Reln*-deficient collecting vessels correlated with increased LYVE-1 expression (Lutter et al., 2012). In none of the previous studies could the contribution of potential secondary and systemic effects be ruled out. Here however, we have found that collecting vessels in *Pdgfb^iLECKO^* mice displayed normal (low) LYVE-1 levels despite the near-complete absence of SMCs. This indicates that SMC contact per se does not lead to downregulation of LYVE-1, which is considered a hallmark of establishment of collecting vessel identity. Together with the inability of ectopic expression of PDGFB in lymphatic capillaries to induce recruitment of SMC, these data suggest that lymphatic capillary- versus collecting-vessel identities are not directly dictated by SMC interaction.

The degree of SMC proliferation during the process of SMC coverage within lymphatic vessels had not been investigated. Our analysis revealed a higher ratio of proliferating SMCs in the ‘younger’ distal collecting vessels than the ‘older’ proximal half, suggesting that proliferation mainly occurs during the collecting vessel maturation process. Although PDGFB also likely contributes to proliferation, the near total absence of SMCs in collecting vessels of *Pdgfb^iLECKO^* shows the strict requirement for PDGFB in the recruitment of the initial pool of SMCs. Furthermore, the reduced coverage seen in the *Pdgfb*^ret/ret^ mice, in which the signalling of the mutant PDGFB is not altered but only its ability to be retained within the local extracellular matrix, further suggests that the initial recruitment is dependent on LEC-derived PDGFB.

Pulsatile contraction of perivascular SMCs in major collecting vessels is known to contribute to the efficiency of lymph drainage (Kunert et al., 2015; Zawieja, 2009). However, whether such contractions are required during normal physiology, or even in pathology, has not been thoroughly assessed. Furthermore the functional impact of a similar contraction in the skin had not been studied. Here, *Pdgfb^iLECKO^* mice displayed severely impaired dermal lymphatic vessel contraction, owing to loss of SMCs, but with no apparent lymphedema and no recordable change in lymphatic drainage in the applied experimental setup (Fig. S3). It should be noted that even genetic mouse mutants that display severe lymphatic vessel defects, such as hypoplasia of lymphatic capillaries in the mouse model of Milroy disease, a form of primary lymphedema (Karkkainen et al., 2001), do not show as severe tissue swelling as humans with the corresponding genetic defect. It is therefore likely that loss of dermal SMCs would have more dramatic consequences in humans.

Ectopic SMC coverage of the lymphatic capillaries has been observed in both primary and secondary lymphedema (Yu et al., 2016), and is postulated to inhibit lymphatic drainage function. In individuals with primary lymphedema, owing to *FOXC2* loss-of-function mutations, the cause of ectopic SMC recruitment was suggested to be a consequence of induction of *PDGFB* expression within the capillaries. Indeed dermal lymphatic capillaries of *Foxc2*^−/−^ embryos displayed abnormal PDGFB expression, but whether this alteration was sufficient or required in this pathology is not clear. Our data show that genetically induced LEC-specific overexpression of PDGFB is not sufficient to drive recruitment of SMCs to lymphatic capillaries. These data, together with the increased SMC coverage in individuals with secondary lymphedema, indicate that other alterations are required in addition to induced PDGFB expression. Our results demonstrate that PDGFB requires the binding to heparan/chondroitin sulfate chains to mediate normal SMC recruitment to the collecting vessels. Whereas collecting vessels have a continuous BM, lymphatic capillaries display only discontinuous BMs and, as shown here, with very low levels of the PDGFB-binding perlecan and collagen IV, thereby potentially limiting PDGFB retention to LECs. Interestingly, *Foxc2*^−/−^ mice display increased deposition of BM components within the defective lymphatic capillaries, which may act together with PDGFB to mediate ectopic SMC recruitment (Petrova et al., 2004). Extracellular matrix alterations may also influence integrin-mediated SMC migration and adhesion, adding to the complexity. In addition the retention motif of PDGFB can be cleaved by proteases, with potential differential abundance or activity in capillaries and collecting vessels. Finally, it is possible that SMC recruitment to lymphatic capillaries is actively inhibited under normal physiological conditions. It has been shown that Sema3-Nrp1-plexin A1 signalling prevents recruitment of SMCs to the valve regions of collecting vessels to ensure normal valve morphogenesis and function (Bouvree et al., 2012; Jurisic et al., 2012); a similar mechanism may exist in lymphatic capillaries.

Precisely how the lymphatic vasculature acquires and maintains its hierarchical structure of capillaries and collecting vessels is not well understood (Schulte-Merker et al., 2011). Here, we demonstrate that the establishment of vessel identity is mainly unaffected by a complete inhibition of lymphatic mural cell recruitment – a process here shown to be strictly dependent on lymphatic endothelial PDGFB expression and pericellular retention. We also clarified that erroneous spatial expression of PDGFB is unlikely to be the single cause of ectopic recruitment of SMCs to lymphatic capillaries in disease. The genetic mouse model of lymph vessel-specific mural cell deficiency provides a novel and specific tool for further studies on the importance of SMCs in lymphatic development and lymphedema.

## MATERIALS AND METHODS

### Mice and treatments

All animal experiments included male and female mice. *Pdgfrb-eGFP* mice [Gensat.org line name: Tg(*Pdgfrb-eGFP*) JN169Gsat/Mmucd] express GFP under the control of the *Pdgfrb* promoter and hence function as mural cell reporter. *Pdgfb-CreER^T2^* and *R26-mTmG* mice have been previously described (Claxton et al., 2008; Muzumdar et al., 2007), and here were combined with inter-crosses to generate *Pdgfb-CreER^T2^, R26-mTmG* mice. To evaluate differential *Pdgfb* expression within the dermal lymphatic vasculature, 4-hydroxytamoxifen (4-OHT) was injected into the abdominal cavity at P14 and mice were sacrificed at P21. Ear samples were then fixed in 4% PFA. *Prox1-CreER^T2^ and Pdgfb^flox/flox^* mice have been previously described (Bazigou et al., 2011; Enge et al., 2002) and were herein crossed with the so-called Ai3 reporter mice [B6.Cg-*Gt(ROSA)26Sor^tm3(CAG-EYFP)Hze^*/J, Stock Number 007903, The Jackson Laboratory, here denoted *R26R-eYFP*] to generate the *Prox1-CreER^T2^*, *Pdgfb^flox/flox^*, *R26-eYFP* (*Pdgfb^iLECKO^*) mice. Recombination was induced from P4 to P7 by oral administration of tamoxifen (20 mg/kg) to the mother. Alternatively, 150 µg of tamoxifen, dissolved in acetone (10 mg/ml), was applied topically to the abdominal skin of the pups. Mice were sacrificed at P21 to analyse the dermal ear vasculature. To analyse mesenteric lymphatic vessels and popliteal lymphatic vessels of the hindlimb at E18.5, recombination was induced from E15 to E17 by injection of 1 mg 4-OHT, dissolved in peanut oil (10 mg/ml) to the mother. To analyse mesenteric lymphatic vessels and popliteal lymphatic vessels at P12, recombination was induced at P1 and P2 by topical treatment of the abdomen with 150 μg tamoxifen. *Pdgfb*^ret/ret^ and *Pdgfb*^ret/wt^ mice have been previously described (Lindblom et al., 2003). Briefly, *Pdgfb*^ret/ret^ mice lack the heparan sulphate-binding domain of PDGFB, following gene targeting. Single-allele knockout mice do not present a phenotype (*Pdgfb*^ret/wt^) and were hence used as controls. Mice were analysed at 10 weeks of age. The *Pdgfb^flox/flox^* mice were also crossed with *Cdh5(PAC)-CreER^T2^* mice (Wang et al., 2010) to generate *Cdh5(PAC)-CreER^T2^*, *Pdgfb^flox/flox^* mice and then crossed with the *R26R-eYFP* mice [B6.Cg-*Gt(ROSA)26Sor^tm3(CAG-EYFP)Hze^*/J, Stock Number 007903, The Jackson Laboratory]*.* In order to specifically delete *Pdgfb* in the mouse endothelium, these mice received 50 μl tamoxifen (20 mg/ml) at P17 and were sacrificed at 4 months.

Animal experiment protocols were approved by the Stockholm North Ethical Committee on Animal Research (permit number N14/13, N168/14) and the Uppsala Ethical Committee on Animal Research (Permit number: C224/12, C225/12, C130/15). All animal experiments were carried out in accordance with their guidelines.

### Whole-mount immunofluorescence staining

Ears and hind limbs were fixed in 4% PFA at room temperature for 2-4 hours and then either stored in PBS with 0.01% NaN_3_ or immediately processed for immunofluorescent staining. To dissect the ear and expose lymphatic vasculature, hair was removed with fine surgical forceps and scissors (Agnthos), and two layers of superficial skin were separated to expose the dermal layer of the ear. Muscles and fat were carefully trimmed away. To isolate the popliteal vessels from the hindlimb of pups at P12, the skin was removed and the exposed blood and lymphatic vessels were dissected together with the underlying muscles. Dissected ears or tissues from hind limbs were then washed with PBS 3×10 min on a rocking table at room temperature. Samples were then blocked in PBS with 1.5% BSA and 0.5% Triton X-100 for 3 h at room temperature followed by addition of primary antibodies and incubation overnight at 4°C. Samples were then washed in PBS with 0.25% Triton X-100 three times, for 1 h each, at room temperature on a rocking table followed by secondary antibody incubation overnight at 4°C. After three washes (1 h each) in PBS with 0.25% Triton X-100 at room temperature on a rocking table, ear samples were flattened on a glass slide and mounted with Prolong Gold (Life Technologies). Mesenteries were fixed in 4% paraformaldehyde at room temperature for 2 h. Samples were washed in PBS, permeabilized in 0.3%-Triton X-100 in PBS (PBSTx) and blocked in 3% BSA in PBSTx. Primary antibody incubation was performed at 4°C overnight, followed by washing in PBSTx and incubation with secondary antibodies at room temperature for 2 h. Samples were then washed in PBSTx and mounted in Prolong Gold for imaging. Primary antibodies included goat anti-podocalyxin (1:400; AF1556, R&D Systems), goat anti-CD31 (1:400; AF3628, R&D Systems), rat anti-CD31 (1:1000; 553370, BD Pharmingen), rabbit anti ERG (1:400; ab92513, Abcam), chicken anti-GFP (1:1000; ab13970, Abcam), mouse anti-α-smooth muscle actin (1:200; α-SMA) (1:250; c6198, Sigma), rabbit anti-PDGFRβ (1:200; ab32570, Abcam), rat anti-PDGFRβ (1:200; 14-1402-82, eBioscience), rabbit anti-PROX1 (1:200; Martinez-Corral et al., 2015), goat anti-NRP2 (1:200; AF567, R&D Systems), rat anti-LYVE-1 (1:400; 0117, R&D Systems) rabbit anti-LYVE-1 (1:400; ab14917, Abcam), goat anti-CCL21 (1:200; AF457, R&D Systems), goat anti-VEGFR3 (1:200; AF743, R&D Systems), rat anti-VE-Cadherin (1:200; 550548, BD Pharmingen), rabbit anti-collagen IV (1:400; 2150-1470, Bio-Rad), rat anti-perlecan (1:200; ab17848, Abcam) and rabbit anti-laminin (1:400; L9393, Sigma Aldrich). Secondary antibodies conjugated with Alexa fluorophores were from Jackson ImmunoResearch Laboratories and Life Technologies (1:300 for staining of mesenteric vessels; 1:400 for staining of other tissues).

### Assessment of SMC proliferation

To assess proliferation of SMCs, mice received three intraperitoneal injections of 5-ethynyl-2-deoxyuridine (EdU, 100 µg/mouse) at P14, P16 and P18, and were sacrificed at P21 for immunofluorescence staining of the ear. EdU staining was performed using the Click-iT EdU imaging kit (Life Technologies) apart from a 3 h incubation of reaction cocktail at room temperature.

### *In vivo* imaging of dermal ear lymphatic vessel contraction

For evaluation of dermal lymphatic vessel contraction, mice were anaesthetized with isoflurane and hair on the dorsal side of the ears was removed using a sharp blade. The head and nose of the mouse was fixed to a customized head holder device and the left ear was glued to a customized plastic plate to prevent movement. TRITC-Dextran (1 µl of 10 mg/ml, 500 kDa, Sigma-Aldrich) was injected subcutaneously with a (30G) insulin syringe (BD Biosciences) or a Hamilton syringe. Mice were then immediately transferred to the imaging stage for time-lapse epifluorescence imaging using either the 20×/1.0 objective on a Leica SP8 laser confocal microscope system (Movie 1, images acquired every 1 s) or a Leica M205FA microscope with a PLANAPO 1.0× objective (Leica Microsystems) (Movies 2,3,4,5, images acquired with an interval of less than 1 s).

### Imaging acquisition of immunofluorescence stained specimens

Confocal images of lymphatic vessels of the ear skin, the popliteal lymphatic vessels or mesenteric lymphatic vessels were acquired using a Zeiss LSM 700 system (Carl Zeiss) with a 20×/0.8 objective or a Leica SP8 laser confocal microscope system (Leica Microsystems) with either of 25×/1.0, 25×/0.95, 20×/0.75 or 10×/0.3 objectives. The images represent maximum intensity projections of *z* stacks that, in the case of overviews, were stitched from multiple tile scan images, either manually using Adobe Photoshop (Adobe) or automatically by the Leica LAF software. Images were processed with FIJI (Schindelin et al., 2012) or Adobe Photoshop software (Adobe). Intensity Adjustments in Fig. 5E′ were applied specifically to the vessel area (dashed line) in order to enhance the visibility of perivascular SMCs. Tiled epi-fluorescence images showing the entire capillary network were acquired using an Axio Observer Z1 system (Carl Zeiss) with a 5×/0.13 objective and images were automatically aligned by Zen blue 2012 software (Carl Zeiss).

### Image analysis

To quantify EdU^+^ cells/mm^2^ SMC surface area, pictures of complete lymphatic collecting vessels were created using manual alignment (Adobe Photoshop) of individual high resolution images. Cells double positive for EdU and α-SMA were manually counted and SMC surface area of a complete collecting vessel was measured using Volocity (Perkin Elmer). To quantify collecting vessel width and SMC coverage, regions with excessive branches and intersecting blood vessels were excluded and vessel width was measured along the vessel and averaged by number of measurements. SMC coverage was quantified using Volocity (Perkin Elmer) (dermal ear skin, popliteal lymphatic vessels) or FIJI (Schindelin et al., 2012) (mesenteric lymphatic vessels) and indicated as percentage of vessel area covered by SMC areas. ‘Capillary density’ was measured using Volocity as area of capillaries in the complete region of interest. Branch points/mm^2^ lymphatic capillaries within a defined region of interest were measured using ImageJ. Lymphatic vessel contraction was analysed in Volocity (Perkin Elmer) and plotted as vessel area against time in GraphPad Prism5 (GraphPad Software).

### Flow cytometry and PCR

Dermal lymphatic endothelial cells were sorted as previously described (Martinez-Corral et al., 2016). Ear skin from adult mice were dissected in ice-cold PBS and digested in collagenase IV (Life Technologies, 10 mg/ml) and DNase1 (Roche, 0.2 mg/ml) in PBS. All digests were incubated for 30-40 min at 37°C, quenched by adding 2 mM EDTA and filtered through a 70 µm nylon filter (BD Biosciences). Cells were washed with FACS buffer (PBS, 0.5% FBS, 2 mM EDTA) and immediately processed for immunolabelling in 96-well plates. Fc receptor binding was blocked with rat anti-mouse CD16/CD32 (eBioscience). Samples were thereafter stained with anti-podoplanin e660, anti-LYVE-1 and anti-CD31 (390) PE-Cy7 (eBioscience). Immune cells and erythrocytes, as well as dead cells, were excluded using anti-CD45 (30-F11), anti-CD11b (M1/70) and anti-TER-119 (TER-119) e450 (eBioscience), together with the cell death dye Sytox Blue (Life Technologies), all detected by the violet laser as one dump channel. For compensation, the AbC anti-rat/hamster compensation bead kit (Life Technologies) was used. Cells were analysed and sorted on a FACSaria cell sorter (BD Biosciences). Single cells were gated using FSC-H/FSC-W and SSC-H/SSC-W followed by exclusion of dead cells, immune cells and erythrocytes in the violet dump channel. (Capillary) LECs were sorted as CD31^+^; podoplanin^+^; LYVE-1^+^ cells. Sorted cells were directly transferred to lysis buffer and mRNA was isolated using the RNeasy Micro Kit (74004, QIAGEN), followed by reverse transcription into cDNA using the iScript cDNA synthesis kit (1708891, Bio-Rad). cDNA was amplified using the TaqMan PreAmp Master Mix Kit (4384267, ThermoFisher Scientific) using TaqMan GeneExpression Assays (ThermoFisher Scientific) of mouse *Cd31* (Mm01242577_m1), mouse *Lyve1* (Mm00475056_m1), and human *PDGFB* (Hs00966522_m1) using the Applied Biosystems 7300 Real-Time PCR system. PCR products were then visualized following electrophoresis in 3% agarose.

### Statistics

Statistical analysis was performed using GraphPad Prism (GraphPad Software) and all differences were determined by unpaired Student's *t*-test, except for the comparison of the number of contraction sites between *Pdgfb^iLECKO^* mice and control mice, for which a Mann-Whitney test was applied. All differences were defined as significant by *P*<0.05. Investigators were not blinded to the group allocation when performing experiments and assessing outcomes.
